# Crack Monitoring of Operational Wind Turbine Foundations

**DOI:** 10.3390/s17081925

**Published:** 2017-08-21

**Authors:** Marcus Perry, Jack McAlorum, Grzegorz Fusiek, Pawel Niewczas, Iain McKeeman, Tim Rubert

**Affiliations:** 1 Department of Civil & Environmental Engineering, University of Strathclyde, Glasgow G1 1XJ, UK; 2 Department of Electronic & Electrical Engineering, University of Strathclyde, Glasgow G1 1XQ, UK; jack.mcalorum@strath.ac.uk (J.M.); g.fusiek@strath.ac.uk (G.F.); p.niewczas@strath.ac.uk (P.N.); iain.mckeeman@strath.ac.uk (I.M.); tim.rubert@strath.ac.uk (T.R.)

**Keywords:** fiber optic sensing, fiber Bragg gratings, concrete cracks, displacement sensors, structural health monitoring, life extension, field trial

## Abstract

The degradation of onshore, reinforced-concrete wind turbine foundations is usually assessed via above-ground inspections, or through lengthy excavation campaigns that suspend wind power generation. Foundation cracks can and do occur below ground level, and while sustained measurements of crack behaviour could be used to quantify the risk of water ingress and reinforcement corrosion, these cracks have not yet been monitored during turbine operation. Here, we outline the design, fabrication and field installation of subterranean fibre-optic sensors for monitoring the opening and lateral displacements of foundation cracks during wind turbine operation. We detail methods for in situ sensor characterisation, verify sensor responses against theoretical tower strains derived from wind speed data, and then show that measured crack displacements correlate with monitored tower strains. Our results show that foundation crack opening displacements respond linearly to tower strain and do not change by more than ±5 μm. Lateral crack displacements were found to be negligible. We anticipate that the work outlined here will provide a starting point for real-time, long-term and dynamic analyses of crack displacements in future. Our findings could furthermore inform the development of cost-effective monitoring systems for ageing wind turbine foundations.

## 1. Introduction

Onshore wind turbines currently supply 10% of Europe’s electricity [1]. As this proportion is only expected to increase [2,3], structural health monitoring is needed to ensure that the infrastructure around wind power remains reliable [4]. On land, wind turbines are typically supported by gravity-based, reinforced-concrete foundations. These foundations can undergo degradation and cracking for numerous reasons, including under-reinforcement during design; shrinkage and thermal gradients during casting; or subsistence and freeze-thaw during service [5]. The sheer diversity of causes suggests that foundation cracking could be common, but, so far, structural health monitoring research has mainly focussed on the rotating and electrical components of wind turbines [6,7]. Foundation monitoring is usually either inferred from models [8,9], or limited to the interface between the foundation and the tower [10,11]. The sparsity of foundation monitoring research is perhaps unsurprising: site surveys suggest that the failure of purely structural elements (the foundation and tower) account for only 1–4% of wind turbine failures [12,13]. It is not prudent to generalise these findings, however, as the root causes of concrete cracks are usually human errors made during planning, design and construction [14]. The repetition of these errors can cause higher failure probabilities to propagate locally over a fleet of turbines. Furthermore, because the impact of a foundation failure is catastrophic, the event poses an increased safety and operational risk [5].

Onshore wind turbine foundations are typically monitored via visual inspections of above-ground surfaces, and in some cases, excavation and more detailed (and costly) inspection of foundation walls (while the turbine is switched off). Neither of these traditional techniques allow underground cracks to be monitored during turbine operation, and they could both be streamlined and improved with the use of modern sensor technology. In other areas of civil engineering, optical fiber sensors such as fiber Bragg gratings (FBGs) have eclipsed the subjective and infrequent nature of inspections by providing quasi-distributed, high-resolution measurements of strains and temperatures in buried concrete structures such as piles, tunnels and foundations [15,16,17]. Retrofitted and surface-mounted sensors in particular have a track-record of detecting strains and crack displacements in existing structures above ground [18,19,20].

In this work, we outline the design, construction, characterisation, installation and verification of FBG sensor modules for monitoring lateral and opening crack displacements of a buried foundation during wind turbine operation. Crack displacement results are correlated with the strains measured by a second series of FBG sensors affixed to the turbine tower, and verified against wind speed and turbine data from the operator. To our knowledge, this is the first time that below-ground cracks have been monitored in a wind turbine foundation during operation. We anticipate that these measurements could be used to better quantify the risks of water ingress and subsequent corrosion of the foundation’s steel reinforcement. Furthermore, by demonstrating that such a real-time monitoring system is feasible, we hope to provide engineers and wind farm operators with a new tool to inform decisions about current asset lifetime, and the design and construction of foundations in future.

## 2. Wind Turbine: Expected Response to Loads

**Figure 1:**
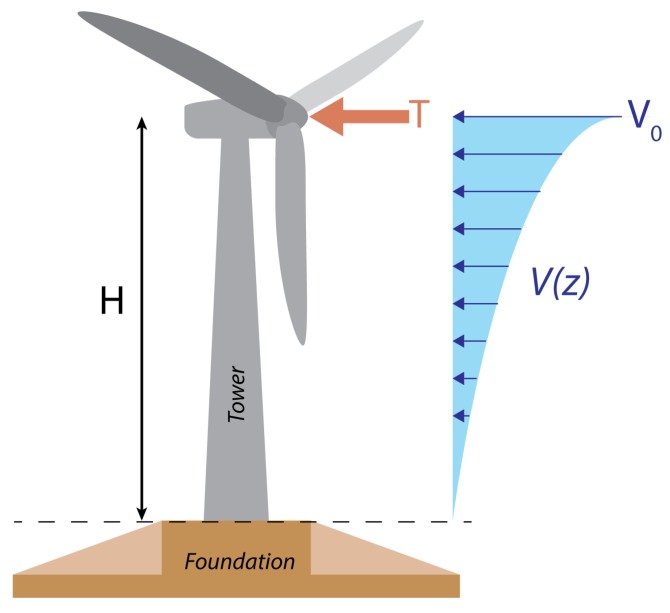
The loads on the wind turbine that contribute to the overturning moment.

The wind turbine monitored in this work consists of a tubular steel tower affixed to an octagonal gravity foundation using prestressing bolts (Figure 1). Sensors for monitoring tower strain and foundation crack displacements cannot be designed or verified without some prior knowledge of the typical response of the wind turbine system to wind loads. This response can be estimated using finite element models [9], but here we opt to use a mathematical approach to aid simplicity and transparency.

### 2.1. Tower Strain

As shown in Figure 1, the wind turbine can be modelled as a fixed-free cantilever, where the overturning moment at the base of the tower occurs due to (i) the thrust acting on the rotor at hub height, *H*; and (ii) the interaction between the tower and a distributed wind profile, V(z) [21]. Under stationary conditions, and for a given wind speed, $V_{0}$, and air density, ρ, the total moment is described by [22]:

(1) $$M=\frac{1}{2}\rho C_{T}AV_{0}^{2}H+\frac{1}{2}\rho D_{T}C_{Fx}\int_{0}^{H}V{\left(z\right)}^{2}zdz.$$

The first component of Equation (1) includes the thrust coefficient, $C_{T}$, which describes the strength of the interaction between the wind and the spinning rotor [23,24]. The parameter, $A=\pi R^{2}$, is the area of the circular disc swept by the turbine blades, each of which has a length *R*.

The second component of Equation (1) describes how the turbine tower of diameter, $D_{T}$, interacts with the wind via a drag coefficient, $C_{Fx}$. During stable wind conditions, the wind profile in the integral can be approximated by [25]:

(2) $$V\left(z\right)=V_{0}{\left(\frac{z}{H}\right)}^{\frac{1}{7}}.$$

Substitution of Equation (2) into Equation (1) reveals a simpler expression for the moment:

(3) $$M=\frac{1}{2}\rho V_{0}^{2}\left(C_{T}AH+\frac{7}{16}D_{T}C_{Fx}H^{2}\right),$$

which can be converted to the maximum strain at the base of the tower using the second moment of area, *I*, and the Young’s Modulus, *E*, of the tower:

(4) $$\varepsilon_{max}=\frac{MD_{T}}{2IE}.$$

**Figure 2:**
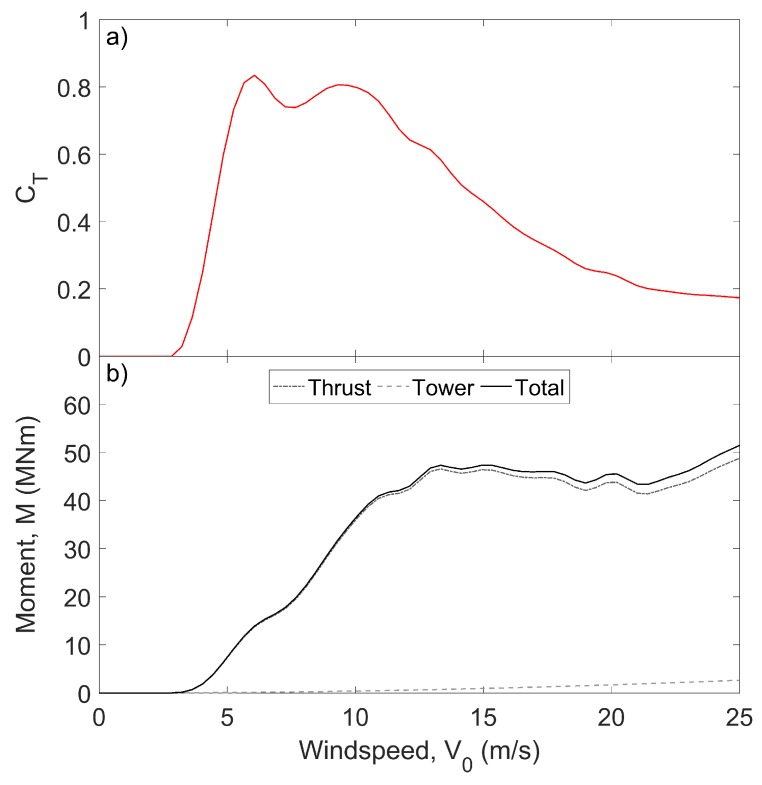
Theoretical values of (**a**) the rotor’s thrust coefficient [26] and (**b**) the overturning moment acting at the turbine base, calculated using Equation (3) and the parameters in Table 1.

Table 1 lists the parameter values used for the wind turbine studied in this work. Substitution of these values into Equation (3) reveals that the overturning moment, plotted for various wind speeds in Figure 2b, rarely exceeds $M=50\times 10^{6}$ Nm. This suggests that the strains measured in the tower should not exceed $\varepsilon_{max}\approx 250$ με.

Note that Equation (4) can be generalised for any wind direction and tower sector as:

(5) $$\varepsilon =\varepsilon_{max}\cos\left(\alpha -\theta_{s}\right),$$

where α is the wind direction (or equivalently, the turbine yaw) and $\theta_{s}$ is the direction that the tower sector of interest faces.

### 2.2. Foundation Crack Displacement

**Figure 3:**
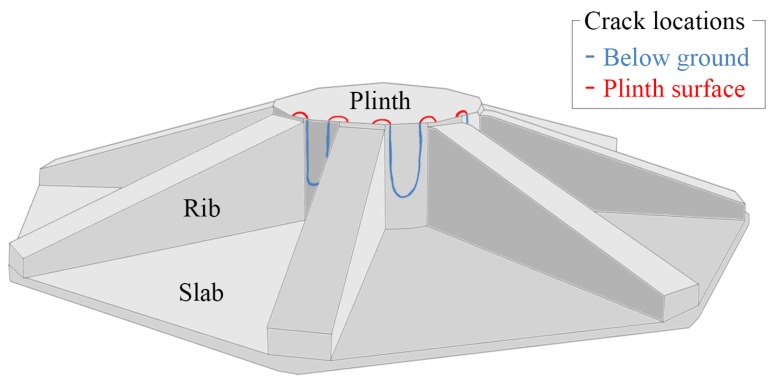
Illustration of the octagonal gravity foundation studied in this work. The cracking pattern that can occur for this design is also shown.

The concrete gravity foundation monitored in this work is of a steel-rib-reinforced, slab design, with a central octagonal plinth (as illustrated in Figure 3). The multiple-stage concrete pour used to construct this particular design can cause thermal cracking to initiate at the interface between the plinth and the ribs. These cracks can, in some cases, be propagated by overturning moments in the prevailing wind direction and by discontinuities in the levels of steel reinforcement. The cracks are minor, but still warrant measurement as cyclic opening and propagation increases the likelihood of water ingress and rebar corrosion.

In general, the sides of the foundation that face the wind are subjected to tensile stresses, causing cracks to open, while the opposite side of the foundation is placed into compression. There should therefore be a correlation between the strains measured in the tower and the opening of cracks. The exact form of this dependence cannot be quantified at this stage, as it could depend on the depth of the cracks, their locations, the degradation of the structure and whether there is an internal network of cracks within the foundation. Measured changes in relative crack displacements could, however, be used to infer a degradation in structural health over time.

## 3. Sensor Design and Characterisation

### 3.1. Fiber Bragg Gratings

**Figure 4:**
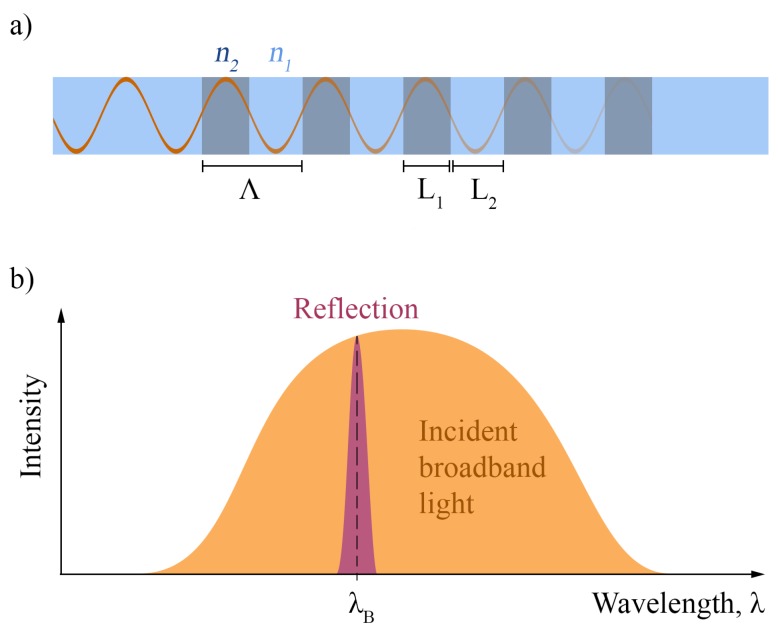
The (**a**) structure and (**b**) reflected spectrum of a fiber Bragg grating. Modulation between high ($n_{2}$) and low ($n_{1}$) refractive index values with a periodicity $\Lambda =L_{1}+L_{2}$ creates partial back-reflections which sum to create a Bragg peak at $\lambda_{B}$.

In this work, fiber Bragg grating (FBG) sensors were used to monitor strains, temperatures and displacements in the structural components of the wind turbine system. As Figure 4 illustrates, FBGs are formed from a 10–20 mm long step-like and periodic variation in the refractive index of a glass optical fiber. When broadband light, guided through the fiber, encounters the FBG, a narrow distribution of wavelengths is reflected back towards the light source. The peak position of this wavelength distribution, termed the Bragg peak, $\lambda_{B}$, shifts when the FBG is subjected to strain, $\varepsilon_{z}$, or temperature changes, ΔT:

(6) $$\frac{\Delta \lambda_{B}}{\lambda_{B}}=k_{\varepsilon }\varepsilon_{z}+k_{T}\Delta T;$$

here, $k_{\varepsilon }=$ 0.78 and $k_{T}=6$ ppm/°C are the strain and temperature sensitivity of the FBG, respectively [28]. To decouple the strain and temperature in Equation (6), it is common practice to place a second, strain-isolated FBG (a thermometer) in thermal contact with each strain sensor [29].

### 3.2. Sensor Fabrication

Three types of FBG sensors were deployed in this work: wind turbine tower strain gauges, FBG thermometers for temperature compensation and custom-built foundation crack-displacement sensor modules. These three sensor types allowed movements in the turbine and foundation to be correlated independently of thermal effects. All FBGs used in this work were of 10 mm length, and were side-written into 20 mm-long, mechanically-stripped sections of acrylate-coated, single-mode optical fiber. The bare fiber sections containing the FBGs were epoxied directly to their relevant packaging or substrate, as this reduces the degradation of strain transfer in the presence of any weakly-bonded acrylate recoating [30].

#### 3.2.1. Strain Gauges and Thermometers

**Figure 5:**
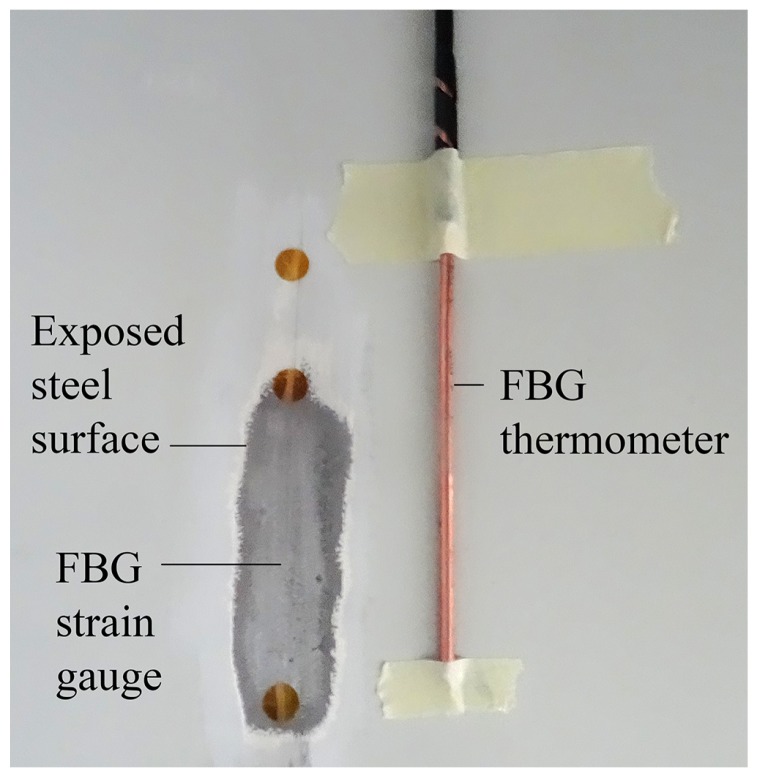
Epoxied strain sensor and adjacent copper-encapsulated temperature sensor prior to adhesive coating and painting.

Strains were monitored near the base of the turbine tower, 50 cm above the plinth, by epoxying bare FBGs to stripped and cleaned steel surfaces on the inside of the tower, as shown in Figure 5. Each FBG strain gauge was installed running along the turbine tower axis, so that the strains caused by overturning moments could be captured. Thermometers were constructed in-house by encapsulating FBGs in two layers of copper capillaries, as outlined in previous work [31]. This provided a strain-isolated reference for the thermal compensation of nearby tower strains.

#### 3.2.2. Crack Displacement Modules

**Figure 6:**
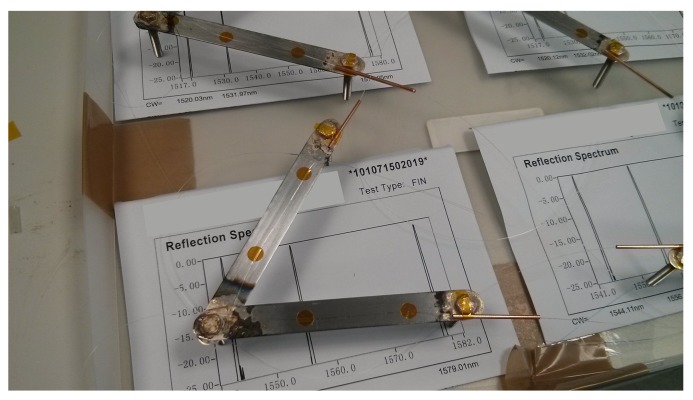
Photograph of foundation sensor module arms with FBGs attached.

**Figure 7:**
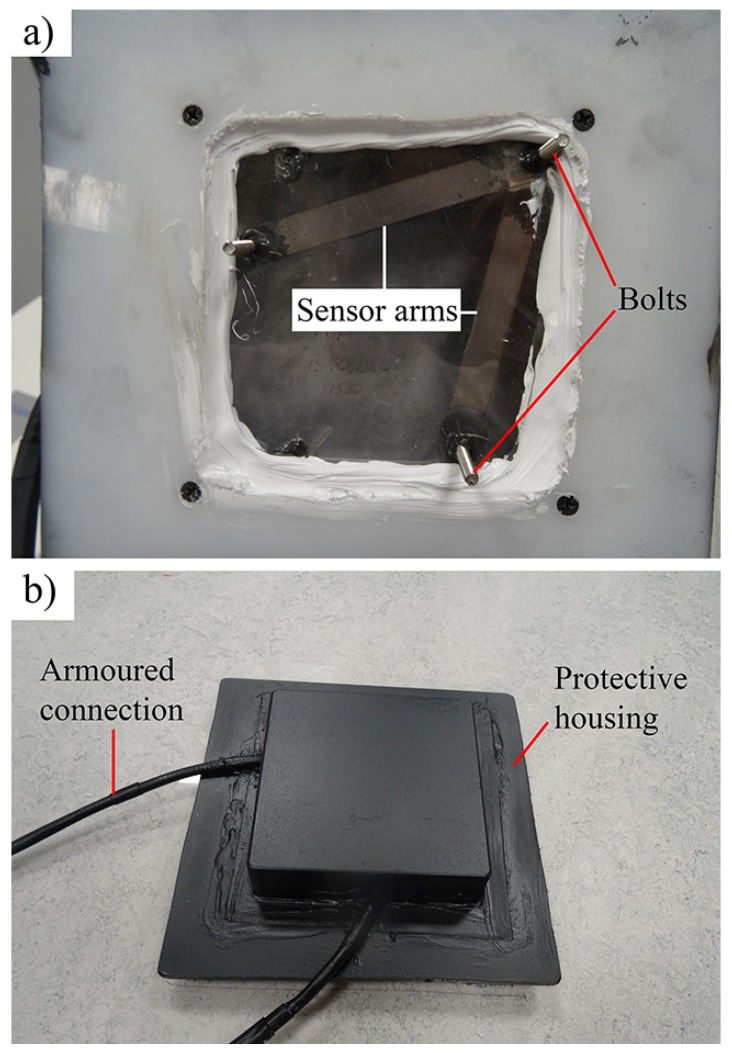
The (**a**) concrete facing and (**b**) soil facing sides of a displacement sensor module, showing the protective housing.

Photographs of crack-displacement sensor modules are provided in Figure 6 and Figure 7. Each bidirectional sensor module allows one crack to be monitored at two nearby locations, and along two directions that are 60° apart. This means that similarities in the response of the two sensor arms can be used to measure crack opening (mode I) displacements with more confidence [32]. Lateral (mode III, shear or tearing) movements in cracks can also be extracted from the *differences* between the two arms (using trigonometry). This design is known to provide reliable measurements of crack displacement, as similar designs have been laboratory tested in our previously published work [33,34].

To fabricate each module, stainless steel bolts (length 25 mm) were inserted through holes in two carbon-steel arms of length $L_{h}=$ 10 cm. These components were joined by induction brazing, with the arms at a 60° angle to each other. After pieces were cleaned of oxidization, a fiber array (made up of an FBG thermometer and two strain sensors) was epoxied to the steel arms. Armoured protection was also affixed to protect the addressing fiber from mechanical damage during installation and service. As shown in Figure 7, the assembly was then housed within an aluminium and acrylic enclosure, with the bolts protruding through a perspex window. Interfaces between materials and the region around the bolts were then sealed in stages using a combination of epoxy, silicone sealant and spray paint. This prevents moisture ingress, which can degrade both steel and glass fiber components [35].

In total, four crack-displacement sensor modules (denoted A, B, C and D) were fabricated. To install each module, three 6 mm diameter holes were drilled either side of a crack in the concrete face of the foundation. The bolts, labelled in Figure 7, were then fixed into these holes using a construction-grade injectable epoxy mortar.

### 3.3. Characterisation

#### 3.3.1. Thermal Response

Thermometers and foundation sensor modules were thermally characterised by heating them within an environmental chamber from 0.5 °C to 30 °C in 5 °C steps. The thermal responses of all sensors lay in the range 11–17 ppm/°C . Note that these thermal coefficients are all higher than for bare FBGs ($k_{T}=6$ ppm/°C ) due to the added thermal expansion of the adjoined steel and copper components. Differences in sensitivity can be attributed to the fact that sensors were manufactured by hand. Once these differences are characterised and known, they do not significantly degrade measurement performance.

FBG tower strain gauges could not be characterised in the lab, as bonding them to the turbine tower has a strong and initially unknown influence on their response (the temperature coefficient is dependent on the tower’s material properties, the quality of the bond and the type of epoxy used). As such, these sensors were characterised during operation, using local air temperatures available from the operator (see Section 5.1). We used a Bayesian approach [36,37], where we made the prior assumption that the tower strain gauges would have a thermal sensitivity of $K_{\pi }=11\pm 0.5$ ppm/°C , i.e., comparable to the FBG components bonded to steel in the lab. Following this, fluctuations in the Bragg peak of the strain gauges were correlated with the operator’s measurements of air temperature using linear regression. This provided real-time observations of thermal sensitivity, denoted $K_{o}$. The prior and observed distributions of sensitivity were then combined to calculate posterior values for strain gauge thermal sensitivity:

(7) $$K_{p}=\Sigma_{p}\Sigma_{\pi }^{-1}K_{\pi }+\Sigma_{p}\Sigma_{o}^{-1}K_{o},$$

where $\Sigma_{\pi }$ and $\Sigma_{o}$ are the covariance matrices of the prior and observed sensitivity and:

(8) $$\Sigma_{p}={\left(\Sigma_{\pi }^{-1}+\Sigma_{o}^{-1}\right)}^{-1}$$

is the covariance matrix of the posterior. Using these methods, the calculated posterior thermal sensitivities of tower strain gauges were typically found to be ≈10 ppm/°C . These sensitivities were then used to compensate for thermal effects on tower strain measurements. Overall, the uncertainty in the strain due to this compensation method was less than 5 με.

#### 3.3.2. Displacement Response of Foundation Modules

FBGs are short-gauge sensors of 10 mm length. This means that they effectively monitor the strain at the centre of each steel arm of a crack displacement module. Each arm, however, is a long-gauge sensor of length $L_{h}=10$ cm, with a strain described by [38]:

(9) $$\varepsilon_{h}=\frac{\Delta L_{h}}{L_{h}}=\frac{1}{L_{h}}\int_{x_{A}}^{x_{B}}\varepsilon_{c}\left(x\right)dx+\frac{1}{L_{h}}\sum_{j}\Delta w_{j}.$$

Surface strains or the opening of existing cracks lead to a relative displacement $\Delta L_{h}$ of the anchoring bolts, which are grouted into the concrete foundation at locations $x_{A,B}$. The strains due to the opening or closing of existing cracks are captured by the integral in Equation (9). These strains are ’predictable’, in the sense that changes in crack displacement are expected to correlate with wind loads and directions. The quantities $\Delta w_{j}$, meanwhile, are a set of added and less predictable strain discontinuities caused by the propagation of new cracks [38]. These new cracks will appear as small, permanent step changes in the strain data: negative or positive depending on whether the cracks occur at or between the anchoring bolts, respectively. In this paper, we focus on the strains caused by changes in existing cracks. Indeed, we have not yet witnessed small, discontinuous changes, but these will be investigated in more detail in future work.

The integral in Equation (9) furthermore shows that each sensor arm provides an average measurement of the concrete strain between its two attachment points, resulting in a reduced strain and spatial resolution. This long-gauge sensing is, however, required in this case because concrete is an inhomogeneous material. Monitoring over small length scales may provide measurements of single crack growth, but long-gauge sensors are essential for monitoring multiple cracks, large cracks and bulk physical properties.

During service, opening displacements in cracks will give rise to analogous ’crack strains’, $\varepsilon_{c}$, which are imperfectly transferred to the sensor module arms and the FBGs (i.e., the strain transfer, $\gamma =\varepsilon_{FBG}/\varepsilon_{c}<1$). Strain losses occur at two interfaces. The first interface is that between the FBG and the steel arm of the sensor module. To ensure strain losses at this interface were low, the strain transfer was calculated from the results of thermal calibration data obtained from Section 3.3.1. As the thermal expansion of silica fibers is negligible, bare FBG temperature sensitivity is primarily a thermo-optic effect (recall that $k_{T}=6$ ppm/°C ) [28]. An FBG bonded to a steel substrate, meanwhile, has an additional contribution from the thermal expansion of the steel substrate, $\alpha_{steel}$ =11 ppm/°C . This means that the strain transfer from the steel to the FBG, $\gamma_{1}$, can be calculated from the measured thermal sensitivity of the overall assembly [39,40,41]:

(10) $$k_{T,\mathrm{measured}}=k_{T}+\gamma_{1}\alpha_{\mathrm{steel}}.$$

Solving Equation (10) for $\gamma_{1}$ revealed that the strain transfer values for all sensor arms were between 0.7 and 0.95. The reasons for this variation are: (i) the sensors were manufactured by hand; and (ii) relatively short (20 mm long) sections of bare fibre were bonded to the steel arms, and this short bonding length amplifies natural variations in workmanship [42].

The second interface that affects crack displacement measurements is that between the bolts and the mortared holes within the concrete. The strain transfer at this interface will be governed by the installation procedures and the mortar used to grout the bolts. Our previous work has shown that similar displacement sensors tested in the lab provided valid and reliable readings of crack displacement [33]. However, unlike the lab experiments in the cited work, the exact strain transfer cannot be quantified directly after field installation. This means that absolute values of crack displacements may be subject to small, variable and unknown errors. These errors will have no impact on monitoring the *relative* changes of crack displacements over time, provided they remain constant over time.

## 4. Field Trial

### 4.1. Sensor Installation

**Figure 8:**
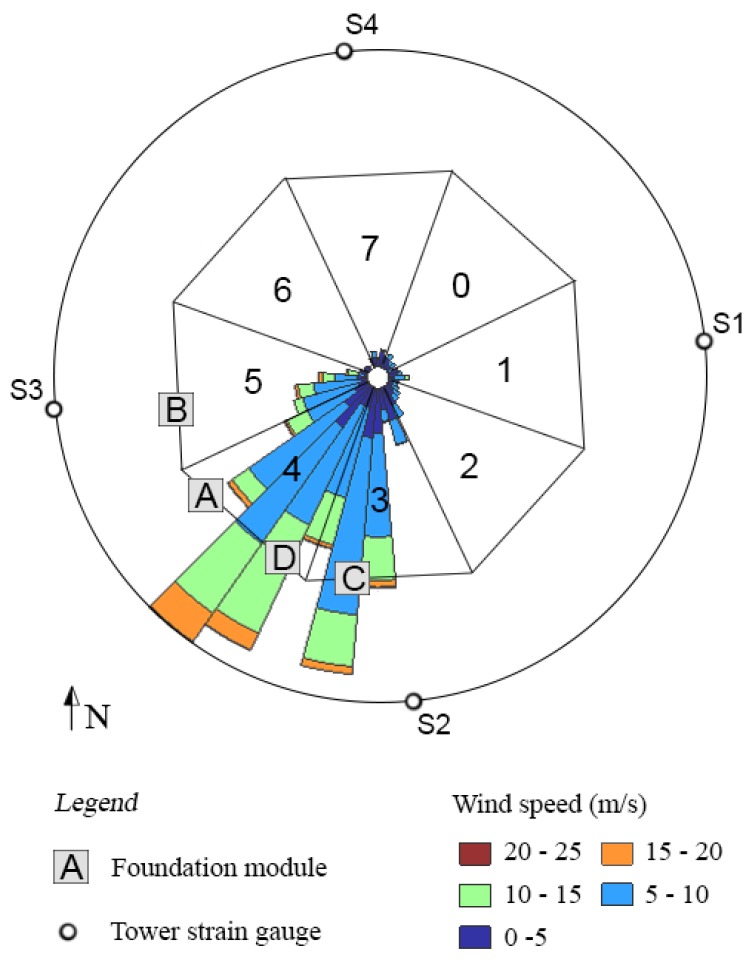
Wind rose for the turbine instrumented in this work. The location of tower strain gauges S (1–4) relative to north is projected onto a unit circle. The octagon represents the directions of the foundation cells (0–7), and the foundation sensor module installation locations are shown (**A**–**D**).

**Figure 9:**
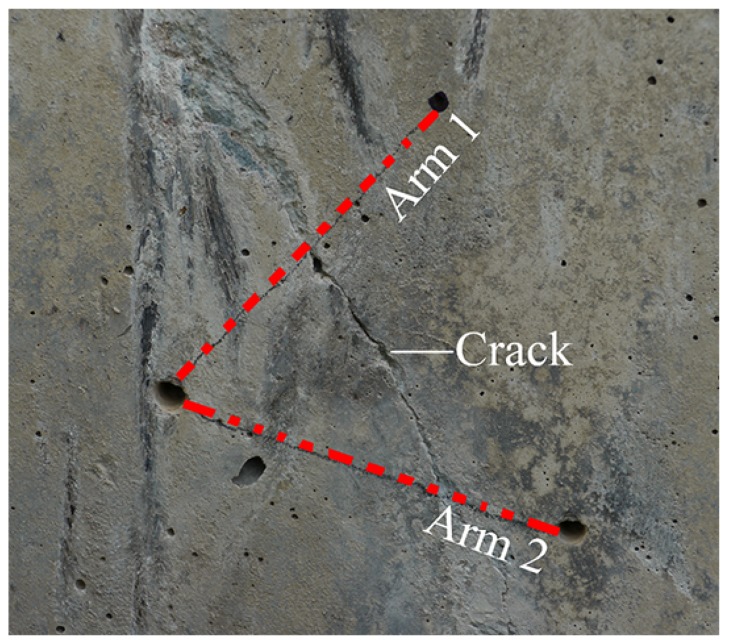
An example of the configuration of the foundation sensor module arms over a crack.

**Figure 10:**
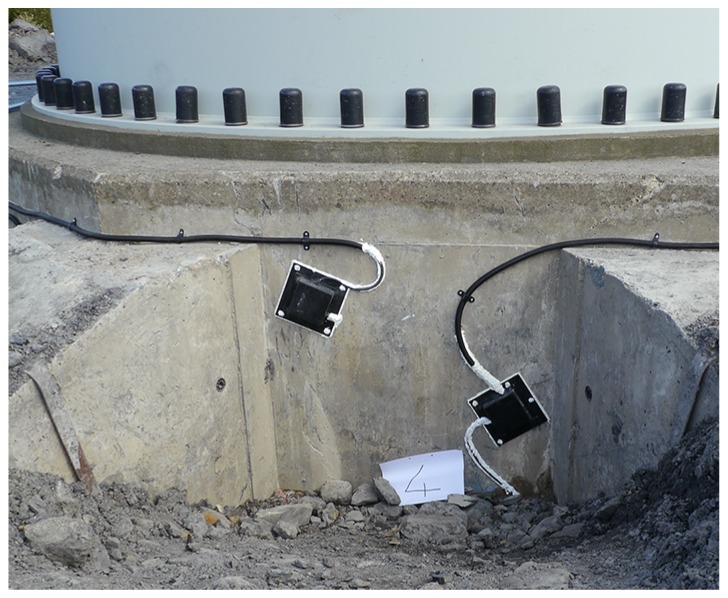
The two completed and sealed sensor modules in cell 4.

For reference purposes, each foundation cell was numbered 0–7, sequentially and clockwise from the entrance door to the tower (see Figure 8). This figure shows the location of the installed tower strain sensors (S1, S2, S3 and S4 and their associated fibre thermometers) and foundation modules A–D. After excavation and inspection of the foundation, it was found that there was visible cracking on cell faces 3, 4 and 5. The wind rose in Figure 8 shows that these cells face the prevailing wind direction. Crack-displacement sensor modules were placed over one crack in cell 3, two cracks in cell 4 (denoted 4L and 4R) and over one crack in cell 5. An example of the orientation of the arms of a sensor module relative to a crack is shown in Figure 9. Two fully installed and hermetically sealed sensor modules are shown in Figure 10.

**Figure 11:**
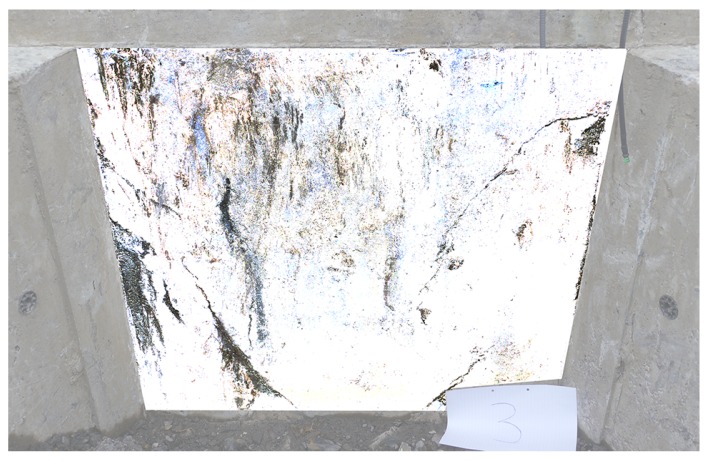
Photograph of cell 3 with image thresholding on vertical face to highlight the location of cracking.

It should be noted that the foundation monitored in this work was in a relatively good condition and presented only minor superficial cracking, as shown in the photograph in Figure 11. This presents challenges for monitoring, as it pushes the sensor technology to monitor foundation cracks even when displacement signals are expected to be small.

### 4.2. Interrogation and Measurement Range

The FBG sensors, at wavelengths between 1520 nm and 1580 nm, were interrogated at a 50 Hz sampling rate. The sensors were serially multiplexed with eight FBGs per interrogator channel. As the Bragg peaks of each sensor cannot be allowed to overlap, the available bandwidth of the interrogator is mainly what defines the allowable measurement range of each sensor. It was assumed that temperatures on site would not exceed the range [−30, +40] °C . Tower strain gauges, meanwhile, were conservatively given enough bandwidth to undergo ±1000 με of strain, while foundation sensor modules could measure ±0.5 mm of crack displacement before spectral overlap. Crack opening displacements exceeding this range were not expected, and would in any case surpass the 5 mε mechanical tensile strength of the FBGs [43].

## 5. Results

At the time of writing, four of the eight installed sensors have been brought on line: tower sensors S2 and S3, and foundation sensor modules B and D. The results in this section are presented for these sensors only.

### 5.1. Temperatures

**Figure 12:**
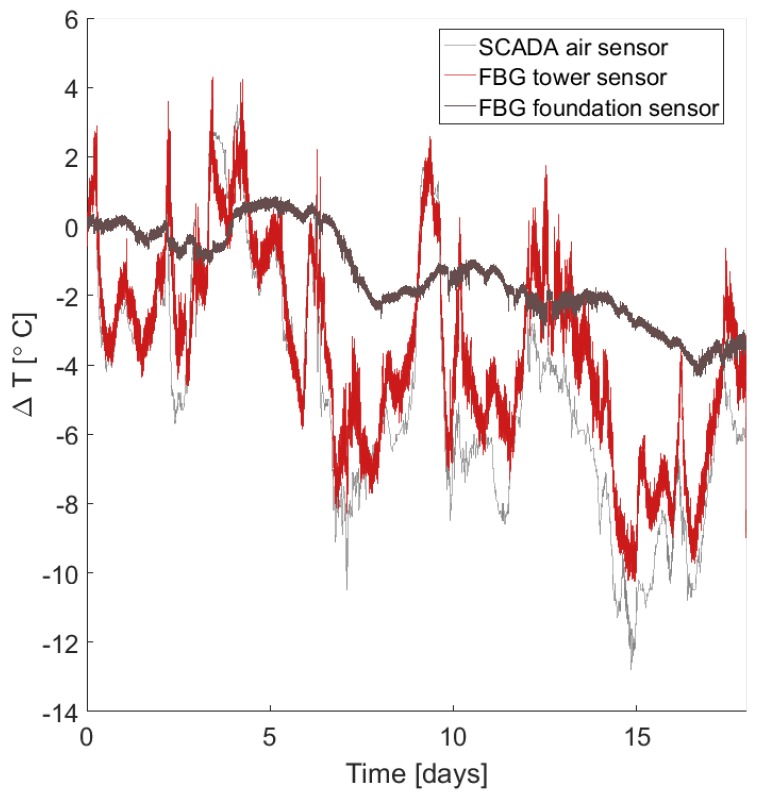
Changes in temperature measured by SCADA air sensors and a FBG thermometers located around the wind turbine tower and foundation.

Figure 12 shows that there is generally good agreement between the temperature changes measured by FBG thermometers and temperature changes measured by the operator’s supervisory control and data acquisition (SCADA) system. Discrepancies between tower and air temperatures may be due to the effects of solar heating of the tower and because air temperatures are measured at a nearby weather tower. Foundation thermometers, meanwhile, match the long-term trends in temperature changes above ground, but undergo thermal damping from the surrounding soil [44].

### 5.2. Tower Strains and Crack Displacements

**Figure 13:**
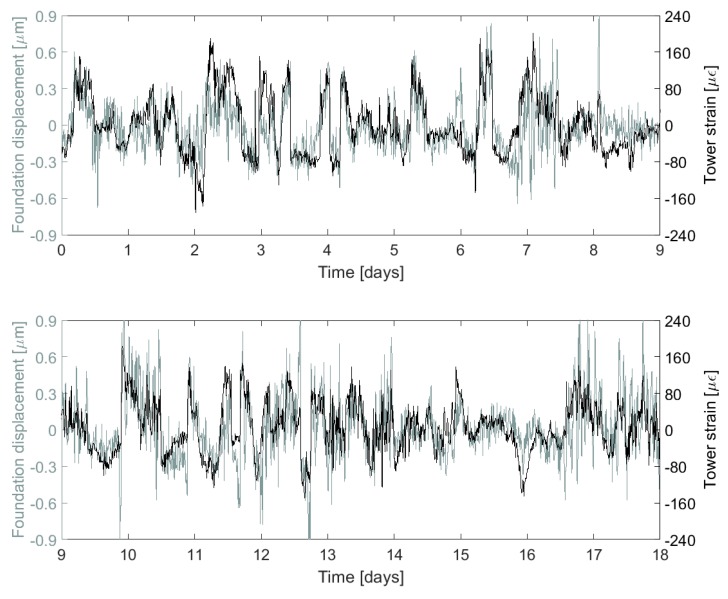
Comparison between (uncorrected) tower strain and foundation crack displacement measurements over two consecutive nine-day periods for sensors S3 and B.

**Figure 14:**
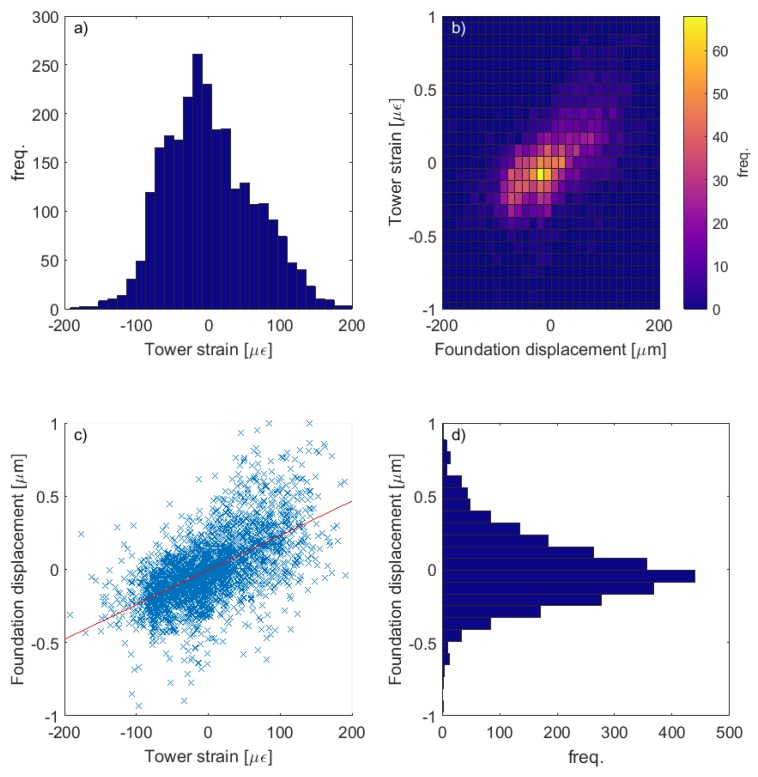
Over the 18-day period shown in Figure 13, histograms of measured (**a**) tower strain; (**d**) foundation displacement; and (**b**) a bivariate histogram of displacement vs. strain. Meanwhile, (**c**) shows a scatter plot showing the correlation (=0.6) between strain and displacement.

**Figure 15:**
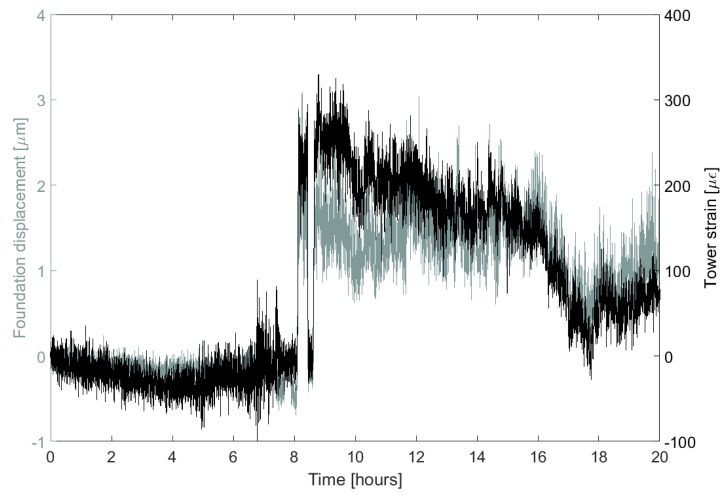
20-hour period showing how transitions to wind turbine operation affect the strains and crack displacements measured by sensors S2 and D.

Figure 13, Figure 14 and Figure 15 show a collection of time series, histograms and scatter plots of the relationships between the measured tower strain and the measured foundation crack opening displacements. The results highlight that the sensors tend to favour positive strains and crack displacements (i.e., tensile tower strain and crack opening). This is a consequence of the fact that these sensors are close to or on the path of the prevailing wind direction.

**Figure 16:**
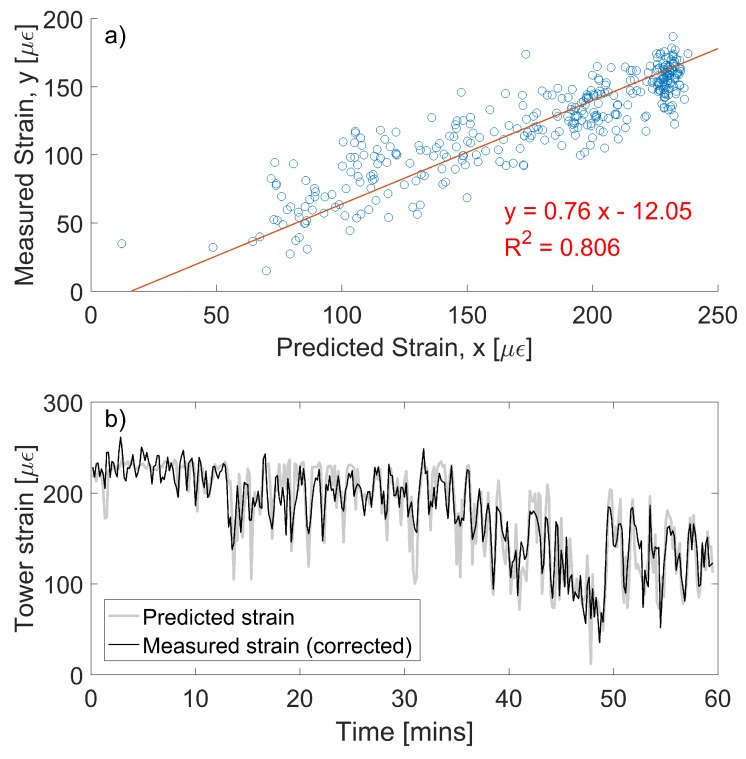
(**a**) the predicted vs. measured strain in the base of the tower during a one-hour window of steady-state, stationary wind conditions and (**b**) time series of predicted strains and measured strains after correcting for offsets and strain transfer.

Note that the comparisons between the model and the strain data outlined in this section can only provide us with an *indication* of the validity of the measurements at this stage. This is because the model used to calculate theoretical tower strain values is simple, and it is based on several assumptions, the most important of which only allows the model to make predictions during stationary operating conditions. Nevertheless, we can see that the measured tower strains, shown for sensor S3 in Figure 13 and Figure 14a, rarely exceed 200 με, an expected result based on the analysis outlined in Section 2. The fact that measured strains are generally lower than the 250 με value predicted in Section 2 could suggest that there is imperfect strain transfer between the tower and the FBG strain gauges. This strain transfer can be estimated if we can predict the theoretical tower strain during stationary wind conditions. Such perfect conditions rarely (if ever) occur in reality, but Figure 16a, shows data that is close to stationary. Here, the strain is predicted using wind speed and yaw data from SCADA, and Equations (3)–(5) . This is compared with the measured strain (after correcting for the effects of wind angle using Equation (5)) for a one hour window which demonstrates near-stationarity. The plot reveals that the strain transfer from the tower to the sensor S3 is around 0.76, and that there is a slight compressive offset in the sensor (likely due to curing and shrinkage of the epoxy). After correcting for these effects, the measured and predicted strain show good agreement, as highlighted by Figure 16b. Some small discrepancies remain, but this is likely because our model is simple: it does not capture dynamic effects, nor does it capture changes in blade pitch or the effects of torque.

Visual assessments of Figure 13 and Figure 15 suggest that the correlations between the measured tower strain and crack opening displacement are reasonable. This is numerically confirmed by the correlation values ≈0.6 (as shown, for example, in Figure 14c). For the two cracks analysed in this paper, the relationship between the tower strain, ε, and the crack displacement, $\Delta L_{h}$, was found to be approximated by the following linear model:

(11) $$\Delta L_{h}=J_{p}\varepsilon ,$$

where the values of parameters are $J_{pA}=0.008$ and $J_{pB}=0.004$ for foundation modules A and B, respectively. The fact that the dependence is close to linear may be a consequence of the fact that crack displacements are so small (displacements do not exceed ±5 μm in either crack). This could indicate that the cracks are only superficial and sub-surface (a conclusion which is backed up by our visual assessments in Figure 11). Figure 14 does make it clear, however, that there is some spread in the data, and some values do depart from the linear correlation shown. This could indicate that a nonlinear crack response is already occuring, particularly at higher loads.

### 5.3. Dynamic Behaviour and Lateral Movement

Figure 15 also highlights interesting dynamic behaviour: sudden increases in tower strain (a consequence of the turbine powering on and off) are transferred into the foundation, causing similar jumps in crack opening displacement. These dynamic events are fairly common, and are responsible for some of the largest tower strains and crack opening displacements. Their behaviour cannot be described by Equation (3), which is only valid for steady-state conditions, but they present a clear signal that can be picked out using a thresholding algorithm. How these events relate to crack opening over the long-term is a question that warrants investigation in future work.

**Figure 17:**
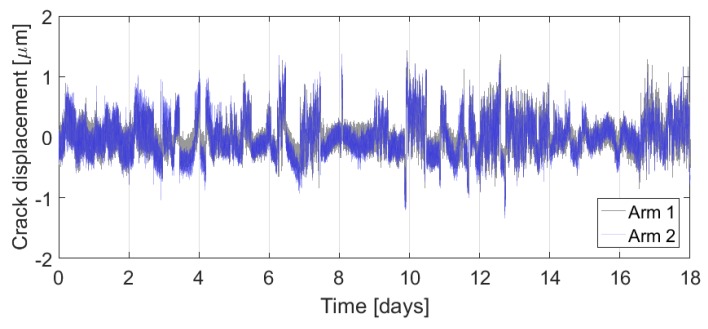
Comparison of displacements measured by both arms of sensor module B.

Finally, Figure 17 shows the overlaid plots of the displacements measured by arm 1 and arm 2 of foundation sensor module B. The responses are comparable, which provides verification of the crack opening measurement. In this example, because the differences between the arms are so small, lateral crack displacements are too small to measure with a high level of confidence. The majority of the movement in the measured cracks is therefore a uniform, mode-I crack opening.

## 6. Discussion

The results presented in this paper have mainly focussed on sensor design, manufacture, calibration, installation and a basic validation of response. The long-term analysis of crack displacements over time, the fusion of sensor data, and verification of dynamic behaviour (wind gusts and sudden changes in wind direction) all represent more significant analyses tasks that will be dealt with in future work. The sensor network installed in this work acquires data at a 50 Hz sampling rate, which is more than enough to capture transient and dynamic effects in wind loads [45]. A full model linking the strain measured to the wind speeds during dynamic conditions in real time could therefore be feasible, but this will require several changes to Equation (3) (outlined in Section 2), which is only valid during steady state, stationary conditions. As more sensors are gradually brought on line, correlation of sensor readouts should provide a clearer picture of the tower and foundation’s stress state. If the same wind loading conditions begin to lead to larger crack displacements, then this may indicate that a crack has degraded. At this point, further consideration will be required to define what levels of crack displacement are indicative of significant damage. More specifically, we will need to decide upon what level of crack displacement allows significant levels of reinforcement corroding agents to penetrate the structure. Such decisions will be made in future work, in part, using the wind turbine operator’s expert knowledge and analysis tools such as logistic regression [46].

The in situ characterisation of the exact strain and temperature response of sensors that are installed in the field remains a challenge in many areas of structural health monitoring. In this work, we have outlined a methodology that we hope provides a template for in-the-field characterisation, at least for a wind turbine system. The advantage of using the Bayesian approach that we opted for here is that as more data becomes available, predictions of the true strain transfers and temperature coefficients can be made with a higher level of confidence. Confidence in the measured tower strains could be further enhanced by using a more detailed model then that presented here. However, we believe it is more preferable to verify validity using more measurements, perhaps thorough further instrumentation of the turbine tower using a combination of conventional and fibre-based strain sensors and accelerometers at various heights, as shown in [47,48]. Recall, however, from Section 3.3.2 that accurate measurements of absolute crack widths would require knowledge of the strain transfer between the bolts of the foundation sensor modules and the mortared holes in the concrete. It could therefore be argued that detailed knowledge of the absolute tower strains is not required for this work, especially if the future goal is to correlate tower strains with *relative* changes in crack width over time. Thus, provided strain transfers in the system do not change over time, these relative changes can be measured. This is why efforts were made to produce robust, hermetically-sealed sensors and use industrial epoxies that are suitable for long-term use in construction.

## 7. Conclusions

This work has outlined the design, characterisation and field installation of fiber-optic sensor modules for monitoring the crack displacements of a wind turbine foundation during operation. A model to verify sensor behaviour under stationary wind conditions has been developed, as have methods that allow in situ characterisation of sensors in the field. This has allowed for more confidence in the measured strain response of the turbine tower, and in turn, more confidence in the measured behaviour of the foundation’s cracks. The results from the sensors interrogated so far suggest that foundation crack opening displacements respond linearly to tower strains and are consistently below ±5 μm. Meanwhile, lateral crack displacements are negligibly small. In future, this work could allow for long-term and dynamic analyses of crack opening to be linked to the penetration rate of water into the foundation. This could inform rebar corrosion models, and allow operators to take informed decisions about asset life extension. The work could also inform the design and construction processes for the world’s growing population of onshore wind turbines.

## Figures and Tables

**Table 1:** Parameter values used to estimate tower strain using Equations (3) and (4).

	Description	Value	Notes
ρ	Air density	1.225 kg/m^3^	
$V_{0}$	Wind Speed	3–25 m/s	Range for rotor cut-in and cut-out [26]
$C_{T}$	Thrust coefficient	0–0.83	Parametric dependence shown in Figure 2a [26]
*R*	Rotor blade length	54 m	
*H*	Hub height	80 m	
$D_{T}$	Tower diameter	5 m	Assumed constant at all heights for simplification
$C_{Fx}$	Tower drag coefficient	0.5	An average value taken from [27]
*I*	Tower second moment of area	2.454 m^3^	$I\approx \frac{\pi }{8}D_{T}^{3}t_{t}$ for thin-walled tubes of thickness $t_{t}$
$t_{t}$	Tower wall thickness	5 cm	
*E*	Tower Young’s modulus	200 GPa	Value for structural steel

## Abbreviations

The following abbreviations are used in this manuscript:

FBG	Fiber Bragg grating
SCADA	Supervisory control and data acquisition
